# Establishment and characterization of HBV-associated B lymphocytes with an immortalization potential

**DOI:** 10.1371/journal.pone.0217161

**Published:** 2019-05-23

**Authors:** Xiaoying Qi, Xien Gui, Ke Zhuang

**Affiliations:** 1 Department of Infectious Diseases, Zhongnan Hospital of Wuhan University, Wuhan, Hubei province, China; 2 ABSL-III Laboratory at the Center for Animal Experiment, State Key Laboratory of Virology, Wuhan University, Wuhan, Hubei province, China; Centre de Recherche en Cancerologie de Lyon, FRANCE

## Abstract

Emerging evidences indicate that hepatitis B virus (HBV) infection is associated with non-Hodgkin lymphoma (NHL), but the mechanisms of HBV-induction lymphomagenesis remain unclear. In this report, retrospective analysis of the prevalence of hepatitis B surface antigen (HBsAg) among NHL cases demonstrated significantly higher HBsAg carrier rate among B-cell NHL cases than controls (other cancers except primary liver cancer) (adjusted odds ratio, 1.56; 95% confidence interval, 1.13–2.16). Furthermore, cells with an immortalization potential existed in the peripheral blood of 4 patients with chronic HBV infection. Characterization of these cells showed their immunophenotypes similar to that of the majority of HBsAg-positive B-cell NHL patients. Immunoglobulin (Ig) gene rearrangements confirmed the clonal Ig gene rearrangements. Cytogenetic analysis revealed abnormal karyotypes of these cells with an immortalization potential. Compared with cells with an immortalization potential that we previously found in B-cell NHL patients by the same way, these cells showed many similar features. In conclusion, cells with an immortalization potential existed in the part of patients with chronic HBV infection before lymphoma development and showed some malignant features. They may be the cellular basis of HBV-associated lymphomagenesis.

## Introduction

Non-Hodgkin lymphoma (NHL) is a common hematological malignancy. About 509,590 new cases of NHL and 248,724 deaths are estimated to have occurred in 2018 worldwide[1]. Mechanisms of NHL development are very complex and the virus infection plays an important role in lymphomagenesis such as Epstein-Barr virus (EBV), hepatitis B virus (HBV), hepatitis C virus (HCV), human immunodeficiency virus (HIV) and herpes virus-8 (HHV-8) [2]. The International Agency for Research on Cancer (IARC) has identified HBV as a risk factor for NHL [3]. Individuals with chronic HBV infection have about 2.8 folds higher risk of NHL than comparison persons [4]. There are estimated about 257 million persons living with HBV infection in 2015 worldwide [5]. HBV infection was endemic in China, where there are 120 million hepatitis B virus carriers and the prevalence rate of HBsAg is 7.2% [6, 7]. A large number of epidemiological studies suggested that hepatitis B virus (HBV) infection was associated with the development of NHL [8–11]. A recent large cohort study has demonstrated the increased risk of NHL in HBV infected patients [12]. Further data suggested that the higher carrier rate of HBsAg was detected in patients with B-cell NHL, but not with T-cell NHL [12, 13]. HBV vaccination was shown to reduce the incidence rate of lymphoma in the teenagers in an endemic area [14]. Antiviral therapy against HBV was found to lead to regression of NHL in HBsAg-positive B-cell NHL patients [15, 16]. The collected evidences from prior trials strongly support an etiologic relationship between HBV and B-cell NHL.

However, the molecular mechanism of HBV-induced NHL development remains unclear. At present, the mechanisms of HBV-mediated Lymphomagenesis have been mainly extrapolated from researches on HBV-induced hepatocellular carcinomas and HCV-mediated Lymphomagenesis [2]. One plausible mechanism is that chronic antigenic stimulation of B-cells promotes B-cell proliferation, increases B-cell DNA damage and thereby leads to malignant transformation of B-cells [17]. In this report, cells with an immortalization potential were found in peripheral blood of the part of patients with chronic HBV infection before NHL development and were characterized. The results indicated that these cells with an immortalization potential have obtained some malignant features similar to cells with an immortalization potential in patients with B-cell NHL and may be associated with HBV-mediated lymphomagenesis.

## Materials and methods

### Prevalence of HBV in non-Hodgkin lymphoma patients

In this study, the prevalence of HBsAg among patients diagnosed histologically with NHL at Zhongnan Hospital of Wuhan University between January 2013 and June 2017 was retrospectively reviewed. Meanwhile, immunophenotypic profiles of HBsAg-positive B-cell non-Hodgkin lymphoma patients were also analyzed. Patients diagnosed with other cancers except primary liver cancer during the same period were enrolled randomly as a control group. All patients were aged ≥16 years.

### Cell cultures

Cases with chronic HBV infection were selected randomly for cell cultures as a study group from outpatient or inpatient at Department of Infectious Diseases of Zhongnan Hospital of Wuhan University between January 2013 and June 2018. All patients were positive for HBsAg, but negative for HIV antibody and HCV antibody. At the same time, healthy volunteers were enrolled randomly as a control group. All healthy people were negative for HBsAg, HCV antibody and HIV antibody. Peripheral blood mononuclear cells (PBMC) isolated from peripheral blood samples of the subjects were cultured in vitro in RPMI 1640 medium supplemented with 10% Fetal Bovine Serum (FBS), 1% Nonessential Amine, 1% HEPES, 1% Glutamine and 1% penicillin/streptomycin at 37°C, 5% CO2. PBMC were cultured for at least 8 weeks. Growth patterns of these cells were observed through the inverted microscope weekly. The study was approved by the institutional ethics committee of Zhongnan Hospital of Wuhan University (Wuhan, China) and patients gave written informed consent.

### Haematoxylin and Eosin staining

The cells with an immortalization potential were attached to slides. Then the slides were stained with Haematoxylin and Eosin. The morphology of the cells was evaluated by the pathologists.

### Immunofluorescence

The cells with an immortalization potential were washed with PBS twice and fixed with 4% polyformaldehyde. After being permeabilized with PBS containing 0.5% Triton X-100 for 20 minutes at room temperature, the cells were blocked with goat serum to prevent nonspecific binding for 30 minutes at room temperature. Then, cells were incubated with the primary antibodies (BD, Franklin Lakes, NJ, USA) overnight at 4°C and followed by the secondary antibodies. Finally, the cells were stained with DAPI for 5 minutes in the dark to visualize nuclei. A fluorescence microscope (Olympus BX53 Microscope) was used to observe for the expression of CD5, CD10, CD20, BCL-2, BCL-6 and IRF-4 (Mum-1).

### Immunoglobulin gene rearrangement analyses

Genomic DNA of these cells with an immortalization potential was extracted using the TIANamp Genomic DNA kit according to the manufacturer’s protocol. The quality and quantity of the extracted DNA were evaluated in AmoyDx spectrophotometer. The monoclonal gene rearrangements of IG heavy chain (IGH), IG kappa (IGK) and Ig lambda (IGL) was detected using the standardized multiplex PCR kit according to the instructions of the manufacturer. Four different IGH multiplex PCR reactions, two different IGK multiplex PCR reactions and one IGL multiplex PCR reactions were respectively utilized to test the clonality of IGH, IGκ and IGλ. Blank control samples, negative control samples (provided by the kit), positive control samples (provided by the kit) and the study samples were run simultaneously each time. The fluorescently labeled PCR products were visualized by capillary gel electrophoresis on the ABI 3500 Genetic Analyzer (Applied Biosystems) and analyzed subsequently by GeneMapper5 software according to their size. The results were evaluated by the pathologist according to the EuroClonality/BIOMED-2 guidelines [18].

### Cytogenetic analyses

After Short-term non-stimulative culture of these cells with an immortalization potential (SIBC 142 and SIBC 173), the cells were treated by 5μg/ml colchicine for 3 hours of metaphase arrest. Giemsa (G) -banding was performed after 0.075 mol/L KCl hypotonic solution treatment and 3:1 methanol acetic acid fixation using standard methods. Karyotypes were analyzed in detail, respectively in 5 cells of case SIBC-173 and in 7 cells of case SIBC-142. Karyotypes were described according to the International System for Human Cytogenetic Nomenclature 2013.

### Virological analyses

HBsAg concentrations in cell culture supernatants were measured by the electrochemiluminescence analyzer (German Roche Cobas-e 411). HBV-DNA of cells with an immortalization potential was measured by Diagnostic Kit for Quantification of Hepatitis B Virus DNA according to the manufacturer’s protocol.

EBV viral load in the cells with an immortalization potential and in plasma of patients with chronic HBV infection was tested by a real-time PCR assay (Bio-Rad MyIQTM 2) using primers for the BamHI W regions of the EBV genome. Genomic DNA of cells with an immortalization potential was extracted using the TIANamp Genomic DNA kit according to the manufacturer’s protocol. The DNA preparation was diluted with 50μl nucleasefree water (Life Technologies) and DNA concentration was computed. Sequences of the forward and reverse primers were as follows: BamHI W forward 5 '- CCCAACACTCCACCACACC-3'; BamHI W reverse 5'-TCTTAGGAGCTGTCCGAGGG-3'. The dual-labeled TaqMan probes ((5'-FAM-CACACACTACACACACCCACCCGTCTC–BHQ -1–3')) for these primers were used in a real-time PCR assay. The EBV gene BamHI W was used to determine EBV viral load in these cells and plasma. Standard curves for the quantification of EBV DNA were created using six-point 10-fold serial dilutions of Namalwa cell (ATCC CRL-1432TM) DNA.

### Full length LMP1 amplification and phylogenetic analyses

Full-length LMP1 was amplified with nest-PCR. PCR was performed on cells derived from patients with chronic HBV infection. The primers used for the first round of PCR were LMP1-F1 (B95-8 coordinate 168012–168032; 5’-TAGAATATGAATGTGGCTTTT-3’) and LMP1-R1 (B95-8 coordinate 169716–169737; 5’-CAAACACACGCTTTCTACTTCC-3’). The second round of PCR was amplified with the LMP1-F2 (B95-8 coordinate 168057–168077, 5’-AGGGAGTGTGTGCCAGTTAAG-3’) and LMP1-R2 (B95-8 coordinate 169584–169603; 5’-ACACTCGCACAGCCCACACC-3’) primers using 1 μl of the first PCR product as the template. The PCR reaction was set up in a reaction volume of 50μl using Primer STAR GC polymerase (TaKaRa). The PCR product was about 1500 KB. Sequencing was performed using the primers LMP1-F2, LMP1-R2 and LMP1-S3 (5’-GGAGGGAGTCATCGTGGTGGT-3’). Multiple alignments of Full-length LMP1 DNA sequences were performed with the CLUSTAL W program [19], using LMP1 from the following 5 reference strains: AG876 (accession no.DQ279927.1), B95-8 Prototype (accession no. V01555.1), B95-8/Raji (NC_007605), China1 (accession no. AY337723.1) and China2 (accession no.AY337724.1). Neighbor-joining phylogenetic trees were constructed using MEGA (version 5.0). Variation of viral LMP1 sequences was determined using the Highlighter tool at the LANL HIV Database (http://hiv.lanl.gov)

### Statistical analysis

Clinical and laboratory parameters of patients in NHL group and control group such as age, sex and HBsAg carrier rates were compared by logistic regression analysis. The odds ratio (OR) and 95% confidence interval (95% CI) for HBsAg carrier rates was estimated by Logistic regression analysis adjusted for age and sex. *P* values <0.05 were considered statistically significant. Statistical analysis was performed using SPSS 13.0 Software.

## Results

### Prevalence of HBV in patients with Non-Hodgkin’s Lymphoma

**Fig 1:**
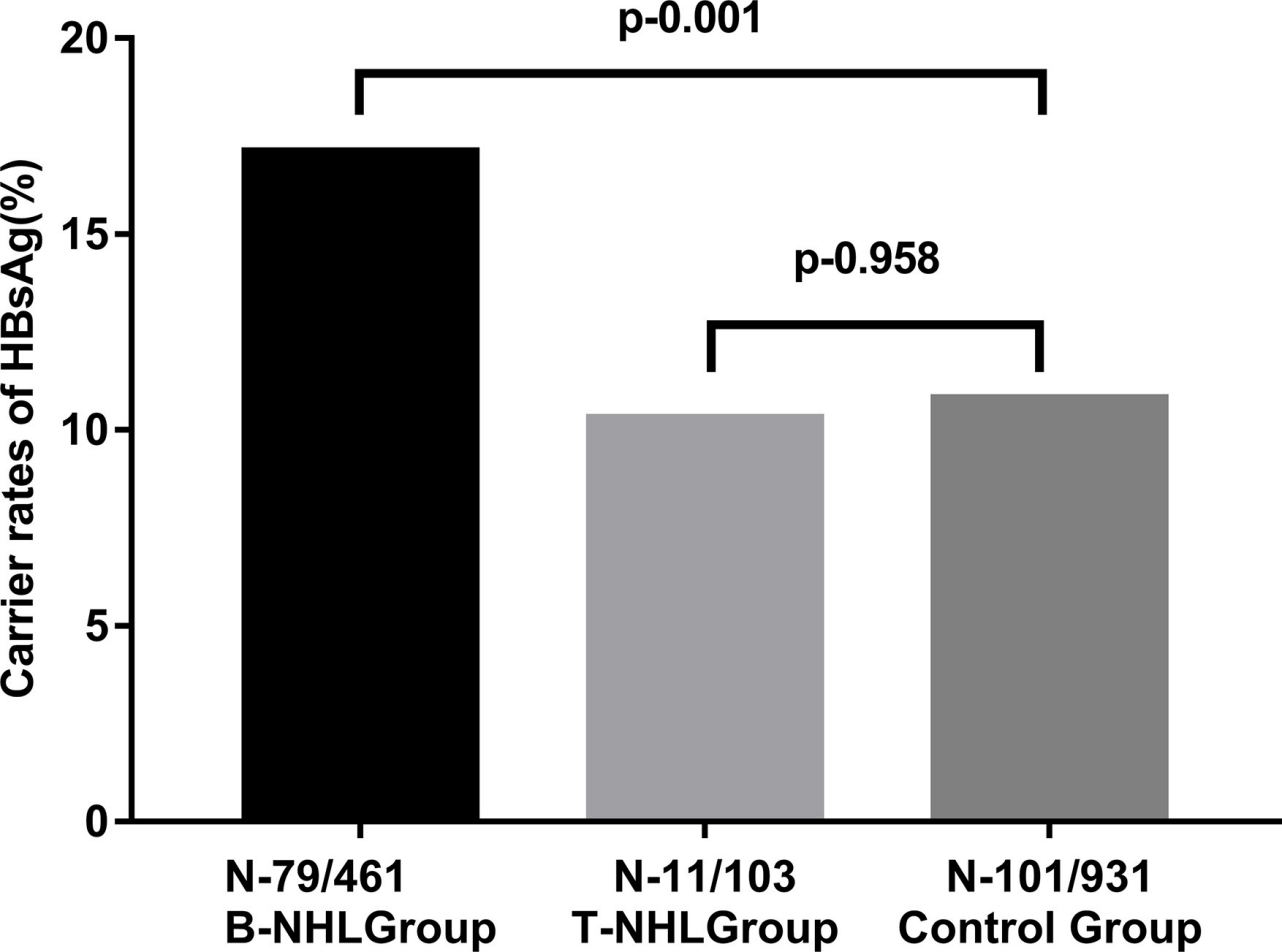
Rates of hepatitis B virus (HBV) infection in non-Hodgkin lymphoma compared with the control group. Carrier rates of HBsAg were significantly higher for the B-cell NHL group compared to the control group (p = 0.001). Carrier rates of HBsAg among T-cell NHL group were comparable to that among the control group (p = 0.958).

597 eligible patients with NHL were enrolled as the NHL group, among which there were 461 cases with B-cell NHL and 103 cases with T-cell NHL. The positive rate of HBsAg in B-cell NHL group and T-cell NHL group was 17.1% (79/461) and 10.7% (11/103) respectively. Meanwhile, 931 patients with other tumor except liver cancer were selected randomly as a control group. The positive rate of HBsAg in the control group was 10.8% (101/931). The rate of hepatitis B virus (HBV) infection in Non-Hodgkin Lymphoma was illustrated in Table 1. Carrier rates of HBsAg among B-cell NHL patients were significantly higher than the control group (10.8%) (*P* = 0.001, adjusted odds ratio, 1.56; 95% confidence interval, 1.13–2.16) after adjusted for sex and age. But, no significant differences of HBsAg positive rate exist between in cases with T-cell NHL (10.7%) and the control group (10.8%) (*P* = 0.958) after adjusted for sex and age (Fig 1).

**Table 1:** Rates of hepatitis B virus (HBV) infection in non-Hodgkin lymphoma compared with the control group.

Factors	NHL group		Control group		*P*	Adjusted OR (95% CI) ^#^
	No. of patients	%	No. of patients	%		
Total NHL patients						
No. of patients	597	100	931	100		
Male	372	62.3	617	66.3	0.114	
Mean age (range)	56.86 (16–96)		61.34 (21–89)		<0.001	
HBsAg-positive	95	15.9	101	10.8	0.004	1.42 (1.04–1.93)
B-cell subtype						
No. of patients	461	100	931	100		
Male	285	61.8	617	66.4	0.102	
Mean age (range)	58.5 (20–96)		61.3 (21–89)		<0.001	
HBsAg-positive	79	17.1	101	10.8	0.001	1.56 (1.13–2.16)
T-cell subtype						
No. of patients	103	100	931	100		
Male	75	72.8	617	66.3	0.181	
Mean age (range)	51.69 (16–77)		61.34 (21–89)		<0.001	
HBsAg-positive	11	10.7	101	10.8	0.958	0.91 (0.46–1.79)

^#^: The OR was adjusted by sex and age

The expression of surface and intracellular markers of 51 patients with HBsAg-positive B-cell NHL is analyzed in detail in Table 2. Bcl-6, IRF-4 (MUM-1) and Bcl-2 were often detected in patients with HBsAg-positive B-cell NHL. But CD10 and CD5 were infrequently expressed in patients with HBsAg-positive B-cell NHL.

**Table 2:** Immunophenotypic features of 51 HBsAg-positive B-cell NHL patients and four cell lines with an immortalization potential.

Antigen	HBsAg-positive, non-Hodgkin lymphoma patients		cell lines with an immortalization potential			
	No. of assessable patients	No. of positive patients (%)	SIBC-62	SIBC-129	SIBC-142	SIBC-173
CD3	51	0 (0)	-	-	-	-
CD5	38	13 (34)	-	-	-	-
CD20	51	51 (100)	+	+	+	+
CD10	49	8 (16)	-	-	-	-
BCL-6	50	35 (70)	+	+	+	+
IRF4	44	35 (80)	+	+	+	+
BCL-2	34	27 (79)	+	+	+	+

+: positive; -: negative

### Establishment of cells with an immortalization potential

**Fig 2:**
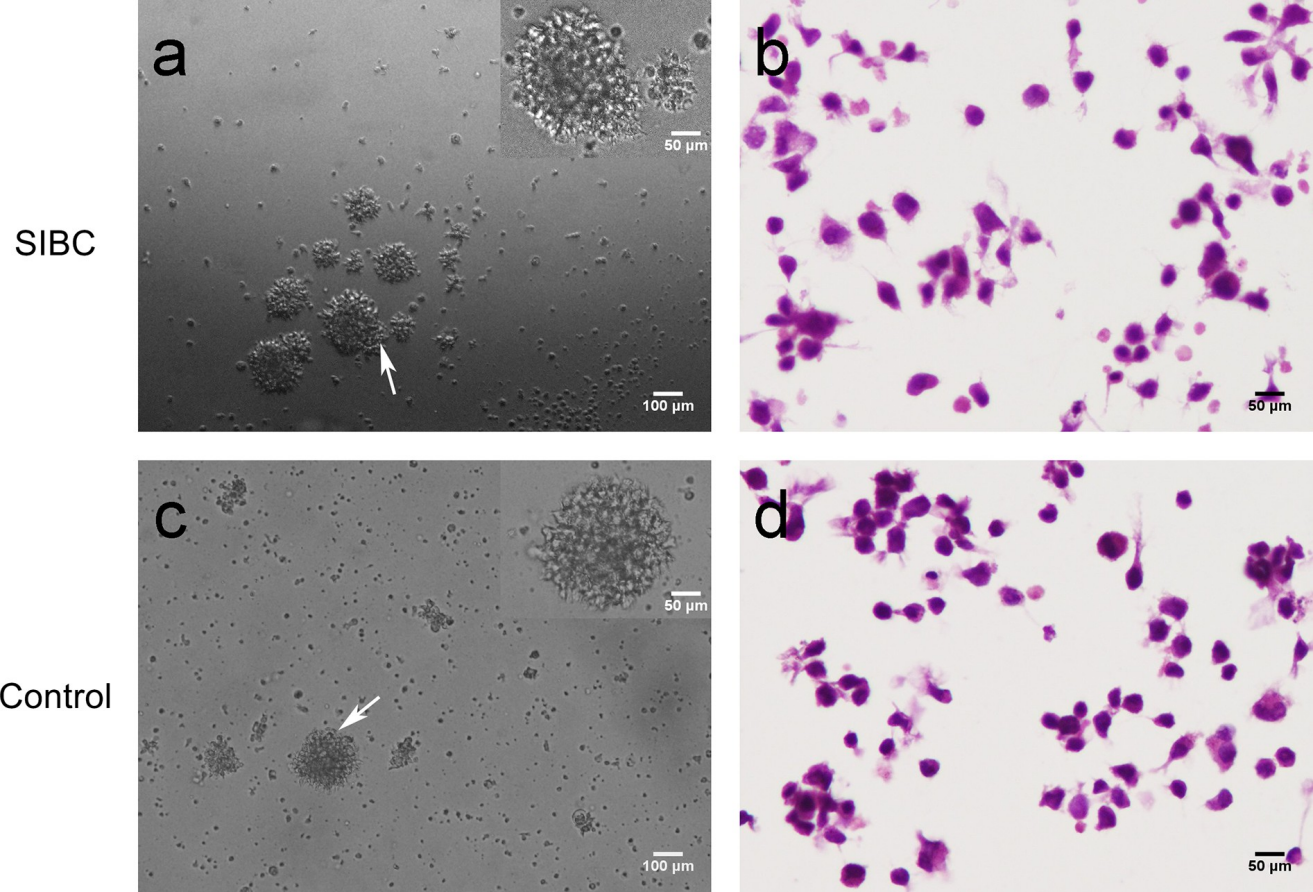
Morphological characteristics of cells with an immortalization potential in the patient with chronic HBV infection compared with cells with an immortalization potential in the B-cell NHL patient. (a) Microscopic image of cells with an immortalization potential in the patient with chronic HBV infection in an inverted light microscope. (b) Haematoxylin and Eosin staining of cells with an immortalization potential in the patient with chronic HBV infection. (c) Microscopic image of cells with an immortalization potential in the B-cell NHL patient in an inverted light microscope. (d) Haematoxylin and Eosin staining of cells with an immortalization potential in the B-cell NHL patient. Insets show white arrow indicating regions at higher magnification.

42 patients with chronic HBV infection were enrolled as the study group. Clinical characteristics of 42 patients with chronic HBV infection were shown in Table 3. Four cell lines with an immortalization potential (SIBC62, SIBC 129, SIBC 142 and SIBC 173) were established from 4 (9.5%, 4/42) cases with chronic HBV infection by cultivating PBMC in vitro. 4 cell lines with an immortalization potential appear after 44 ~ 56 days of culture. They were a few at the beginning and increased gradually, without exogenous stimulus. The cells grew in clumps in vitro. Their growth patterns in vitro were similar to that of cells with an immortalization potential from B-cell NHL patients (Fig 2). But none of cells with an immortalization potential were established from healthy volunteers (32 cases). The morphology of the four cell lines was accessed with Haematoxylin and Eosin staining. The cell lines were composed of small to medium-sized cell and the majority of them were small lymphocytes. The abnormal nucleoplasm ratio and no typical nucleoli were detected in the medium-sized cell. These cells showed some degree of atypia, but less marked atypia than the cells with an immortalization potential (SIBC 14) from B-cell NHL (Fig 2). No difference was detected between the morphology and growth patterns in vitro of the cell line from the HBeAg-negative patient and that of cell lines from the HBeAg- positive patients. The four HBsAg-positive patients with cells with an immortalization potential were followed for more than a year and failed to be detected for lymphoma. These results indicate that these cells with an immortalization potential have acquired the ability to proliferate autonomously and were possibly associated with HBV-induced B-cell NHL.

**Table 3:** Characteristics of HBsAg-positive patients enrolled for cell culture.

Clinical characteristics		Cases with cells with an immortalization potential		Cases without cells with an immortalization potential	
		No. of patients	%	No. of patients	%
No. of patients		4		38	
Male		4	100%	19	50%
Mean age (range)		32.75 (25–39)		35.79 (21–59)	
HBeAg-positive		3	75%	24	63.2%
Serum HBV DNA-positive		4	100%	19	50%
Serum EBV Viral Load (log_10_ Copies/500ng DNA, mean ± SD)		2.00 ±2.31		2.59 ±2.32	
cells with an immortalization potential	HBV DNA-positive	0	0%		
	EBV DNA-positive	4	100%		

+: positive; -: negative

### Immunophenotype of cells with an immortalization potential

**Fig 3:**
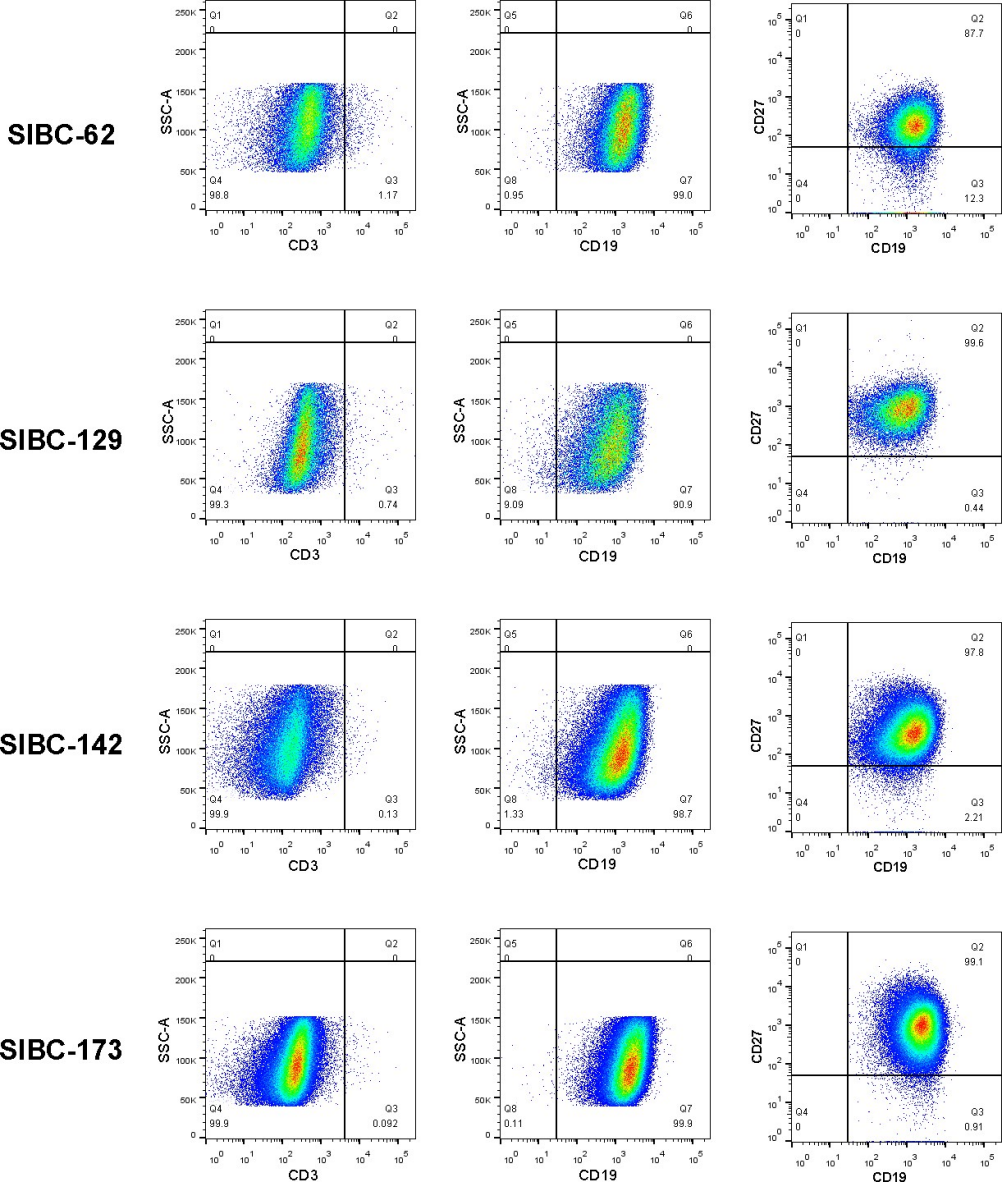
Flow cytometry analysis of the cells with an immortalization potential. Antigen expression patterns of the four cell lines with an immortalization potential (SIBC62, SIBC129, SIBC142 and SIBC173) were determined by flow cytometry with antibodies against CD3, CD19 and CD27. These cell lines were negative for CD3 and positive for CD20 and CD27. The major population in the four cell lines was CD20+CD27+ memory B cells. The number indicates the percentage of cells in the marked gates.

**Fig 4:**
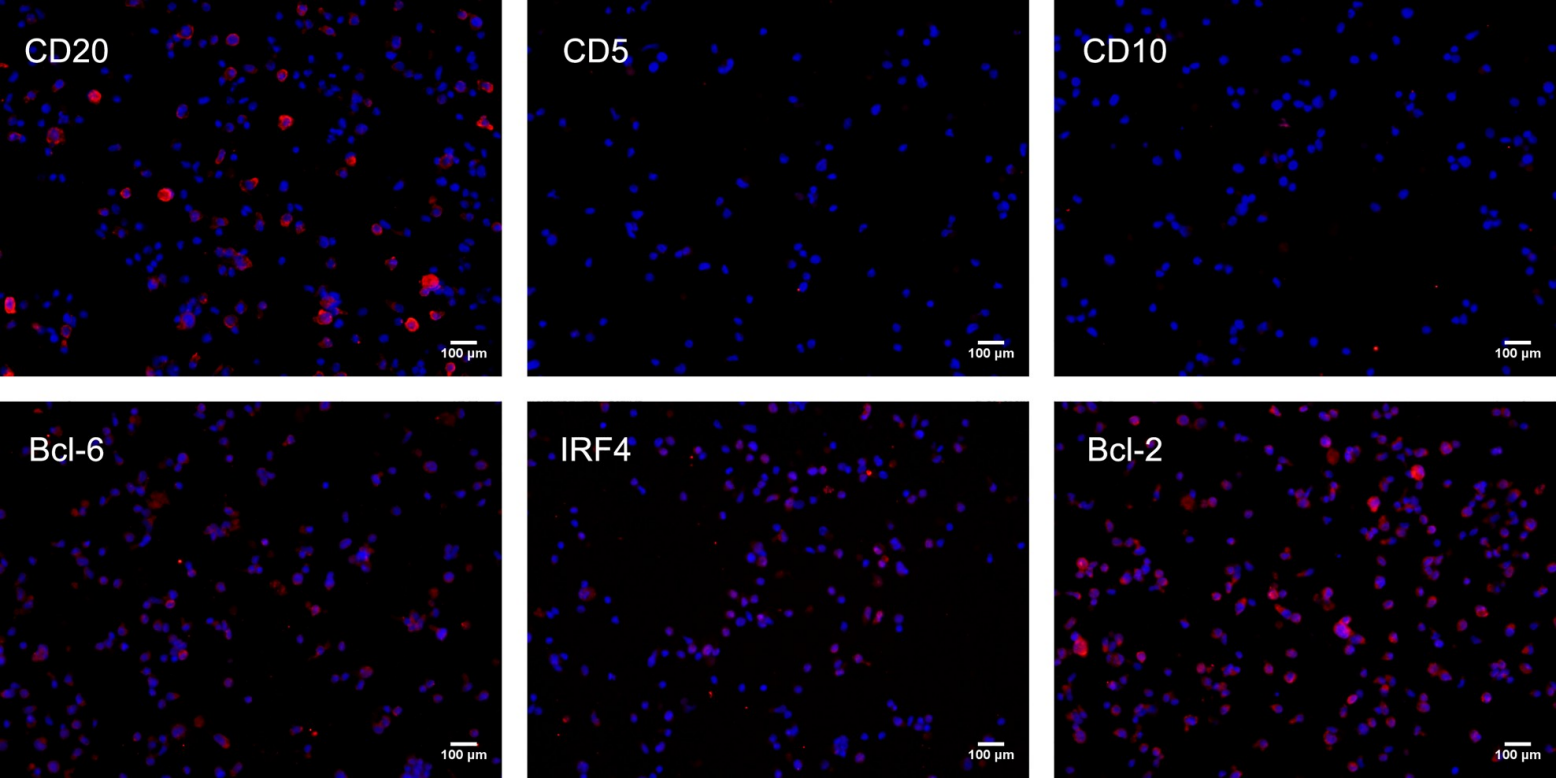
Immunofluorescence analysis of the cells with an immortalization potential. The cells with an immortalization potential were positive for CD20, BCL-2, BCL-6 and IRF-4 (shown in red) and were negative for CD5 and CD10. Nuclei (shown in blue) are counterstained with 4′, 6-diamidino-2-phenylindole (DAPI). Scale bar: 100 μm.

The immunophenotypes of the four cell lines with an immortalization potential were further investigated. The expression of CD19 was observed on more than 95% of cells and CD3 on less than 5% of cells by flow cytometry. Besides, the majority of the cells with an immortalization potential are CD19+CD27+ cells (Fig 3). The finding suggested that these cells were of B-cell origin. Moreover, the cells were found to be positive for CD20, BCL-2, BCL-6 and IRF-4 (Mum-1) and be negative for CD10 and CD5 by Immunofluorescence (Fig 4), which was similar to the immunophenotypic profiles of the majority of HBsAg-positive B-cell NHL patients (Table 2). The immunophenotypes of the cell line generated from the HBeAg-negative patient was the similar to others from the HBeAg- positive patients. These data indicated that the cells with an immortalization potential and the majority of HBsAg-positive B-cell NHL possibly shared similar patterns of differentiation according to immunophenotypic profile.

### Clonality of cells with an immortalization potential

**Fig 5:**
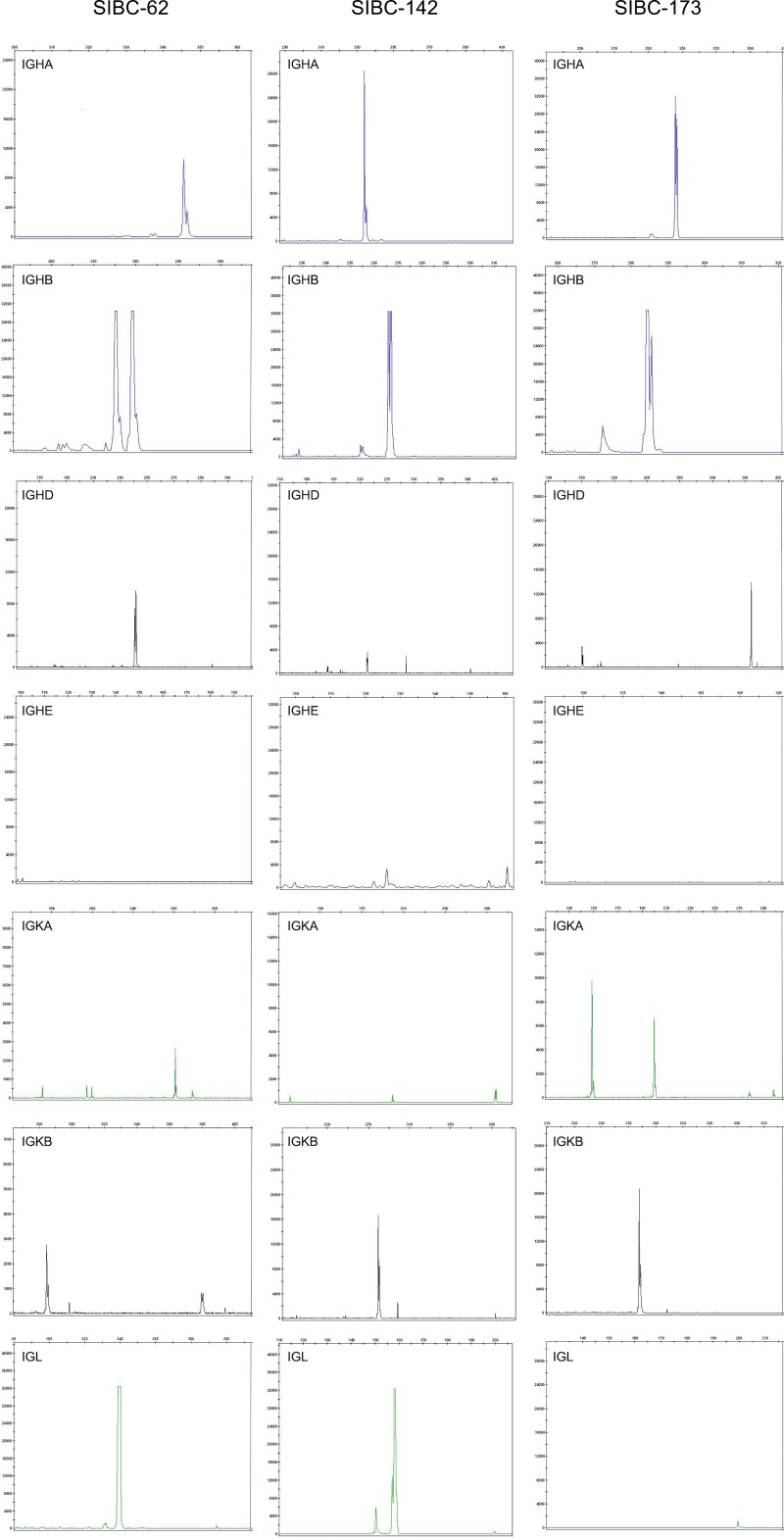
Results of the BIOMED-2 PCR assay for detection of clonality of cells with an immortalization potential. One or two clonal peaks were detected in the three cell lines (SIBC62, SIBC142 and SIBC173).

Clonality of the four cell lines with an immortalization potential were evaluated by Ig gene rearrangement technology (IGH and light chain IGK/IGL). These cell lines presented one or two reproducible clonal peaks (Fig 5), demonstrating that these lines were monoclonal. The findings indicated the nature of the cell lines from their monoclonal origin.

### Cytogenetics of cells with an immortalization potential

**Fig 6:**
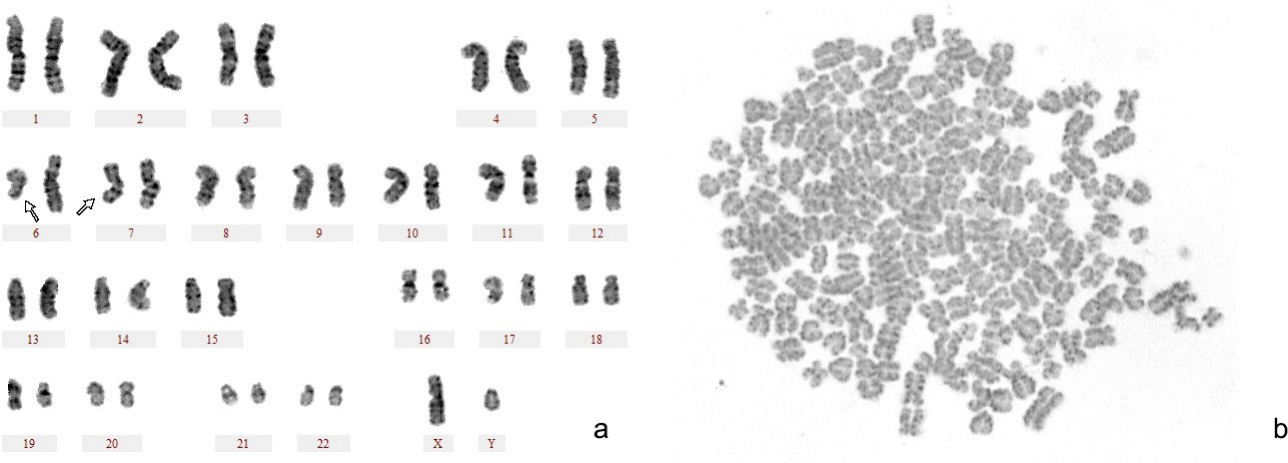
Karyotypic abnormalities of the cells with an immortalization potential in patients with chronic HBV infection. (a) Structural aberration of chromosome. The white arrow depicts a chromatid break (b) the number aberrations of chromosome. The number of chromosomes was 92.

Cytogenetic analysis of the cell lines with an immortalization potential (SIBC 142 and SIBC 173) showed abnormal karyotypes. Karyotype of SIBC-173 was interpreted as 46,XY [5], del (7) (q32) [3], del (6) (q23) [1], del (14) (q32) [1], del (6) (q23) [1]. Karyotype of SIBC-142 was interpreted as 46, XY [7]. But SIBC-142 showed the abnormal chromosomes numbers. The numbers of chromosomes ranged from 46 to 92 (Fig 6). The findings suggested that the cell lines obtained clonal aberrations of chromosomes.

### HBV and EBV status in cells with an immortalization potential

Cells with an immortalization potential were shown to be negative for HBsAg and HBV DNA and positive for EBV DNA. EBV viral load in cells with an immortalization potential and in plasma of patients with chronic HBV infection was illustrated in Table 3. Although EBV viral load was detected in cells with an immortalization potential, EBV viral load in plasma of Cases with cells with an immortalization potential was comparable with that in plasma of Cases without cells with an immortalization potential.

**Fig 7:**
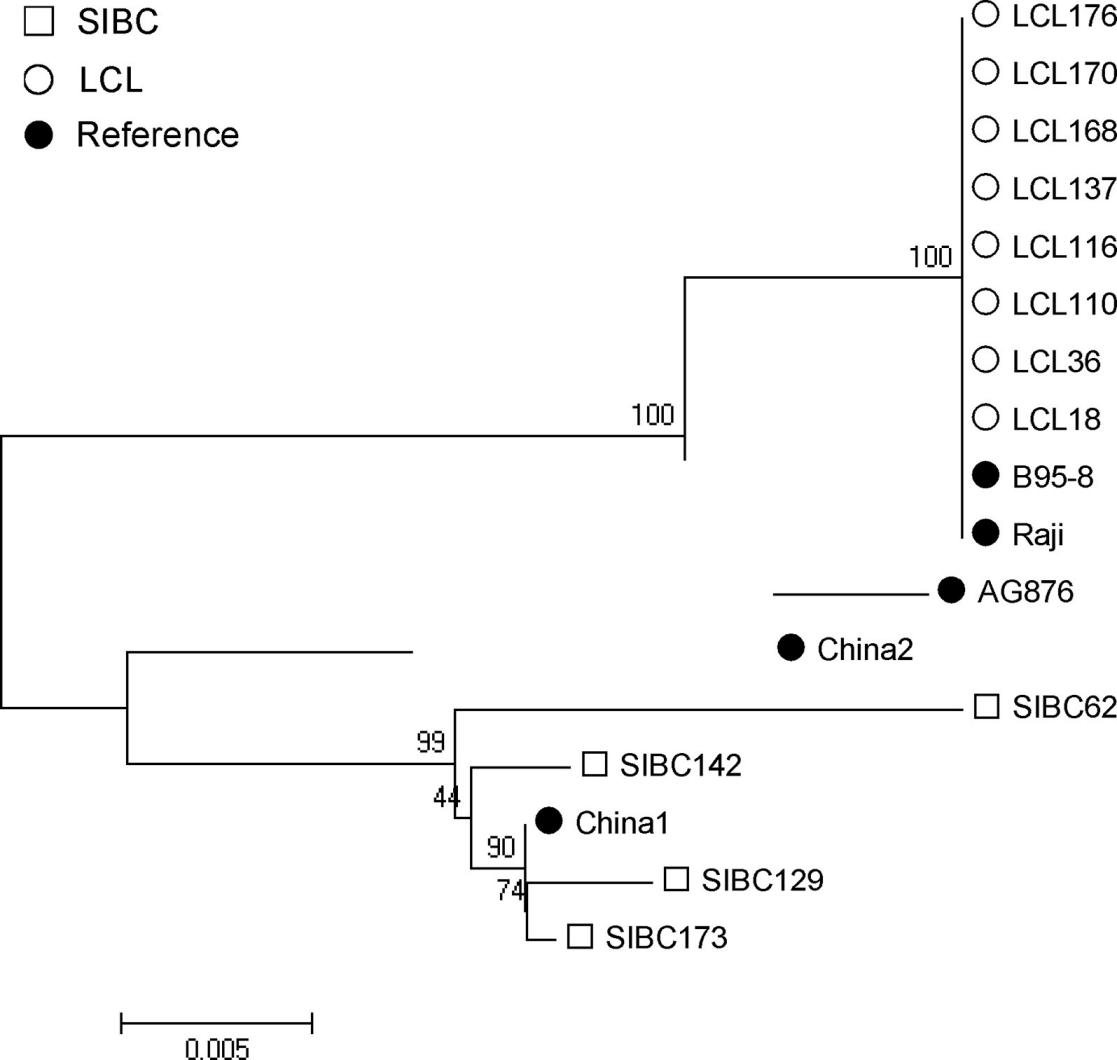
Neighbor-joining phylogenies of LMP1 DNA sequences from EBV in cells with an immortalization potential. SIBC and LCL indicate the HBV- associated cell lines with an immortalization potential (SIBC62, 129, 142 and 173) and LCL lines, respectively. Genetic distances representing the number of nucleotide substitutions per site between LMP1 sequences (0.005) are indicated at the bottom of the tree.

In order to better understand the evolutionary origin of EBV subtypes in HBV- associated cells with an immortalization potential and determine the common and distinct DNA alterations in viral LMP1 genomes of the HBV- associated cells with an immortalization potential, full length LMP1 sequences were amplified from 4 cell lines with an immortalization potential (SIBC62, 129, 142 and 173) and 8 LCLs. The Neighbor-joining system evolutionary tree is constructed based on a reference LMP1 gene sequence of B95-8, China1, China2, AG876 and Raji. Phylogenetic and highlighter plot analysis showed that the LMP1 of cells with an immortalization potential in chronic HBV infection was clustered closely with the China 1 strain instead of the China 2 strain, which were both isolated from Chinese nasopharyngeal carcinoma tissues. But LMP1 sequences from LCLs clustered closely with reference strains of B95-8 and Raji. The Highlighter plots showed an imbalance among the LMP1 gene sequences of the four cell lines with an immortalization potential (Fig 7). These results indicated that EBV genotypes in cells with an immortalization potential from chronic HBV infection were highly homologous to EBV in Chinese nasopharyngeal carcinoma tissues, but there was a certain diversity of EBV LMP1 gene sequences among cell lines with an immortalization potential from different patients.

## Discussion

B-cell non-Hodgkin lymphoma (B-cell NHL) is the most common subtype of NHL and occurs at a higher incidence among chronic HBV infection individuals. However, HBV’s contribution to B-cell NHL has remained elusive. Prior studies have documented the positive relationship between HBV and NHL. But the increased risk of NHL among people infected with HBV was reported to be different in endemic areas and non-endemic areas and HBV seemed to play a different role in various subtypes of NHL [8–12]. To know the association between HBV and B-cell NHL in the local region, we retrospectively analyzed the HBsAg carrier rate among the NHL patients in the local region. We found the higher positive rate of HBsAg in patients with B-cell NHL than patients with the other cancer, suggesting that HBV possibly acted as an etiologic factor in B-cell malignant transformation. But we did not detect this positive relationship between HBsAg carrier and risk of T-cell NHL, indicating that HBV infection does not induce T-cell NHL development. This result was consistent with prior epidemiologic studies [8, 10].

To further provide experimental evidences, we cultured in vitro PBMC from patients with chronic HBV infection and found that novel cells with an immortalization potential existed in peripheral blood of the part of chronic HBV infection patients without lymphoma. These cells were confirmed to be of B-cell origin by showing positive staining for CD19, negative staining for CD3 and the monoclonal result of the Ig gene rearrangement. But cells with an immortalization potential were not established from healthy volunteers. These data strengthen the assumption that HBV possibly was associated with B-cell NHL.

At present, B-cell lines with an immortalization potential were reported to be established from PBMC of patients with Leukemia or lymphoma [20–24]. Studies of these cell lines have facilitated understanding of hematological malignancies [23]. In this study, we established the novel cells with an immortalization potential from chronic HBV infection patients before the development of lymphoma. Undoubtedly, characterizing these cell lines is useful to understand the mechanisms of HBV-induced NHL development. In consequence, biological features of these new B lymphocyte lines were found to be similar to that of the cells with an immortalization potential derived from B-cell non-Hodgkin lymphoma (NHL) in many respects.

Cells with an immortalization potential derived from patients with chronic HBV infection appeared approximately after 5–7 weeks in culture and grew in clumps like the cells with an immortalization potential derived from patients with B-cell NHL that we established. Time of appearance and growth patterns of these cells with an immortalization potential in vitro was also similar to that of cell lines established from B-cell NHL in the same way by prior studies [25].

To compare immunophenotypes of cells with an immortalization potential with HBV- associated B-cell NHL, we selected cell surface markers that were frequently tested clinically in HBV- associated B-cell NHL patients. The cell surface marker analysis of the cells demonstrated that they expressed CD20, BCL-2, BCL-6 and IRF-4 antigens, but lacked expression of CD10 and CD5. The immunophenotypic profiles of cells with an immortalization potential were similar to that of the majority of HBsAg-positive B-cell NHL. The result further supported the association between the cells and HBV-induced NHL development. Furthermore, differentiation patterns of cells were evaluated according to some antigens related to the differentiation profiles. CD20 antigen is a marker of mature B lineage cells [26]. MUM1/IRF4 is a marker indicating post germinal center (GC) cells and expressed in a small percentage of GC cells differentiating to plasmacyte or memory cell and in plasma cells [27]. It is involved in terminal B cell differentiation and lymphocyte activation [28]. CDl0 and Bcl-6 are markers of GC cells [29]. Thus the cells possibly originated from post GC cells.

Monoclonicity is an important feature of lymphoma, which discriminate lymphoma from reactive lymphoproliferations. EuroClonality Consortium has established the BIOMED-2 multiplex PCR system to standardize evaluation of clonally rearranged Ig and T cell receptor (TCR) genes. Prior studies have demonstrated that the BIOMED-2 multiplex PCR system showed high sensitivity and specificity of the detection of clonal rearrangements [30–32]. The BIOMED-2 system has been widely used clinically in the diagnosis of lymphoid malignancies. In the present study, the BIOMED-2 multiplex PCR system was used to analyze immunoglobulin gene rearrangements of cells with an immortalization potential. The cells all showed immunoglobulin gene monoclonal rearrangement like cells with an immortalization potential from B-cell NHL. The result suggested that the cells possibly derived from the clonal expansion of a common precursor like lymphoma.

The cytogenetic aberration is another important feature of lymphoma. Chromosomal analysis of these cells with an immortalization potential revealed that cytogenetic aberrations were often seen in cells. Deletion of the long arm of chromosome 7 (7q) of these cytogenetic aberrations was frequently observed in mature small B-cell lymphoid malignancies [33, 34]. 7q32 deletion was a characteristic feature of splenic marginal zone lymphomas (SMZL) and has the diagnosis and antidiastole value of splenic B-cell lymphomas [35]. In addition, this deletion was reported to be possibly involved in an early event of in the development of lymphoplasmacytoid tumor [36]. The cells with an immortalization potential existed in patients with chronic HBV infection were shown to be possibly mature small B-cells with lymphoplasmatic features according to their morphology and immunophenotypes. Thus 7q32 deletion was possibly also involved in the development of the part of cells with an immortalization potential. But detailed molecular cytogenetic mapping is required to investigate the role of 7q32 deletion in the development of the cells and HBV-associated NHL.

To understand the role of HBV in the formation of cells with an immortalization potential, the amount of HBsAg in the supernatant of cells and HBV DNA in cells were measured. But unexpectedly, HBsAg and HBV DNA was not detected in these cells. Likewise, HBV DNA was reported not to be detected in B-cell NHL tumor cells [37]. These data suggested that the development of cells with an immortalization potential was possibly not linked with directly integrating of HBV DNA into the host genome. The possible mechanism of these cells development may be that chronic antigenic stimulation might cause sustained activation and proliferation of B-cells, predisposing to DNA damage, cytogenetic aberrations, overexpression of proto-oncogenes and transformation into SIBC. JIAN BO and coworkers demonstrated that HBsAg was able to promote cell viability and to reduce apoptosis in the human peripheral lymphoblastoid cell line, IM-9 through the SIRT1‑NF‑κB signaling pathway [38]. Another research found that CDR3 sequences of Ig exhibited a significant homology to HBsAb in HBsAg positive DLBCL, suggesting that HBV-associated DLBCL might originate from HBV antigen-selected B cells [39]. In addition, although EBV DNA was detected in cells with an immortalization potential, EBV viral load in plasma was comparable between cases with cells with an immortalization potential and cases without cells with an immortalization potential. Therefore, EBV might not play the key role in these cells development. Effect of HBV coinfection with EBV on NHL development and interaction between HBV and EBV need further to explore.

## Conclusions

The novel cells with an immortalization potential existed in the part of patients with chronic HBV infection before the development of NHL. These cell lines share similar features with NHL and possibly are associated with HBV-induced lymphomagenesis. The cell lines provide useful models for exploring HBV-induced lymphomagenesis.

## Supporting information

S1 Fig Growth patterns in vitro. Four cell lines with an immortalization potential from the patient with chronic HBV infection grow mainly in clusters. In the upper row the pictures of invert light microscopy (magnification 20×), In the lower row the pictures of invert light microscopy (magnification 40×) are shown.
(TIF)

S2 Fig Morphologic characteristics. Morphologic characteristics of SIBC-62, SIBC-129, SIBC-142 and SIBC-173 are shown by Haematoxylin and Eosin staining.
(TIF)

S3 Fig Immunophenotype. The immunophenotypes of SIBC-62, SIBC-129, SIBC-142 and SIBC-173 are shown by Immunofluorescence.
(TIF)

S4 Fig Clonality. The Clonality of SIBC-62, SIBC-129, SIBC-142 and SIBC-173 are shown by Ig gene rearrangement.
(TIF)
