# Supplementary figures and images for: Establishment and characterization of HBV-associated B lymphocytes with an immortalization potential
Source: PLoS One. 2019 May 23;14(5):e0217161. doi: 10.1371/journal.pone.0217161 (PMC6533042; doi:10.1371/journal.pone.0217161)

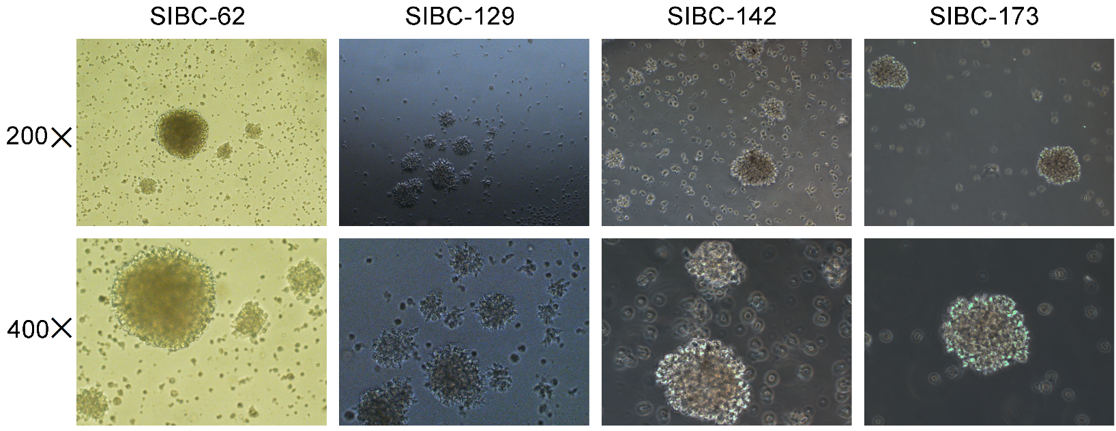

Supplement: S1 Fig — Four cell lines with an immortalization potential from the patient with chronic HBV infection grow mainly in clusters. In the upper row the pictures of invert light microscopy (magnification 20×), In the lower row the pictures of invert light microscopy (magnification 40×) are shown. (TIF) [file pone.0217161.s001.tif]

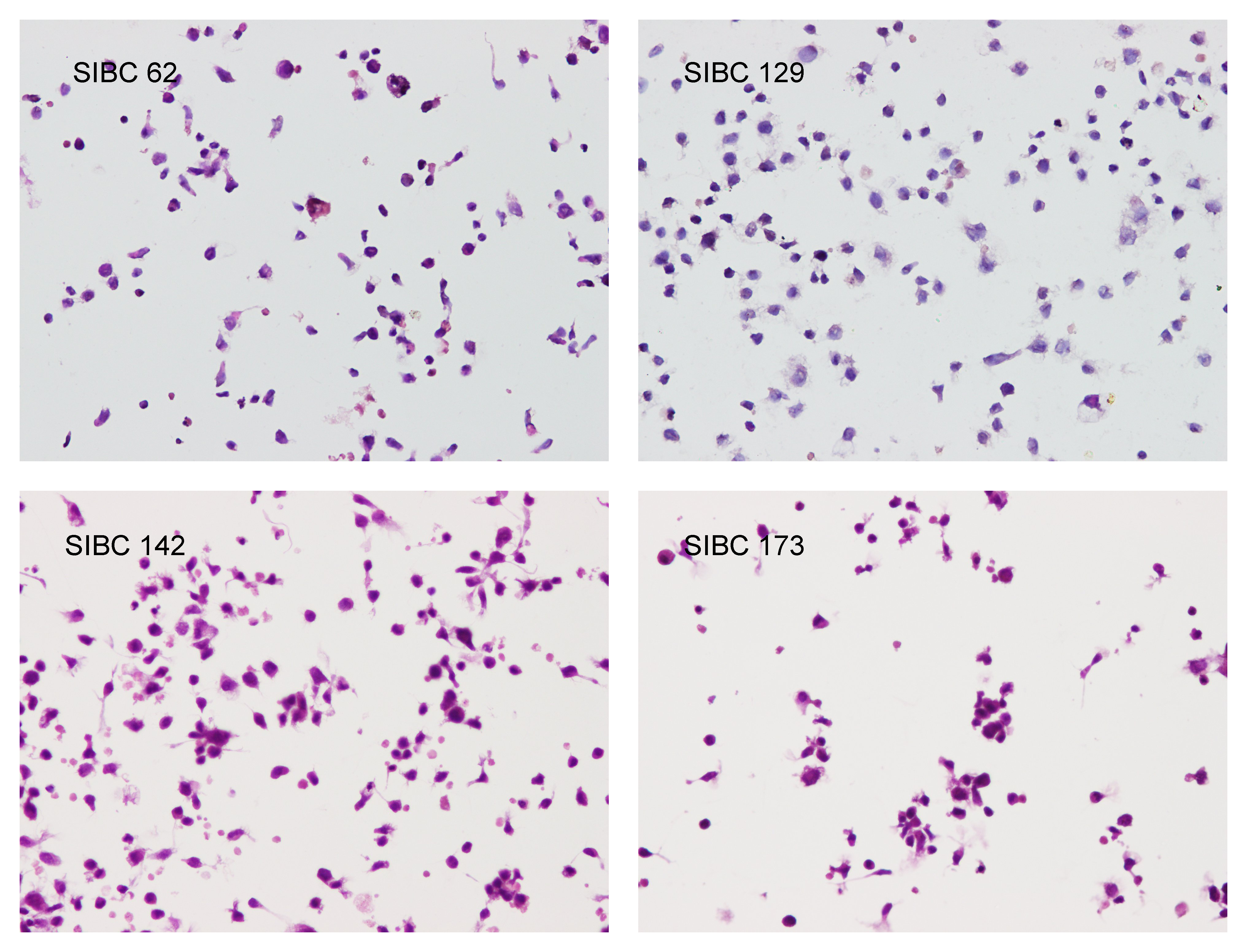

Supplement: S2 Fig — Morphologic characteristics of SIBC-62, SIBC-129, SIBC-142 and SIBC-173 are shown by Haematoxylin and Eosin staining. (TIF) [file pone.0217161.s002.tif]

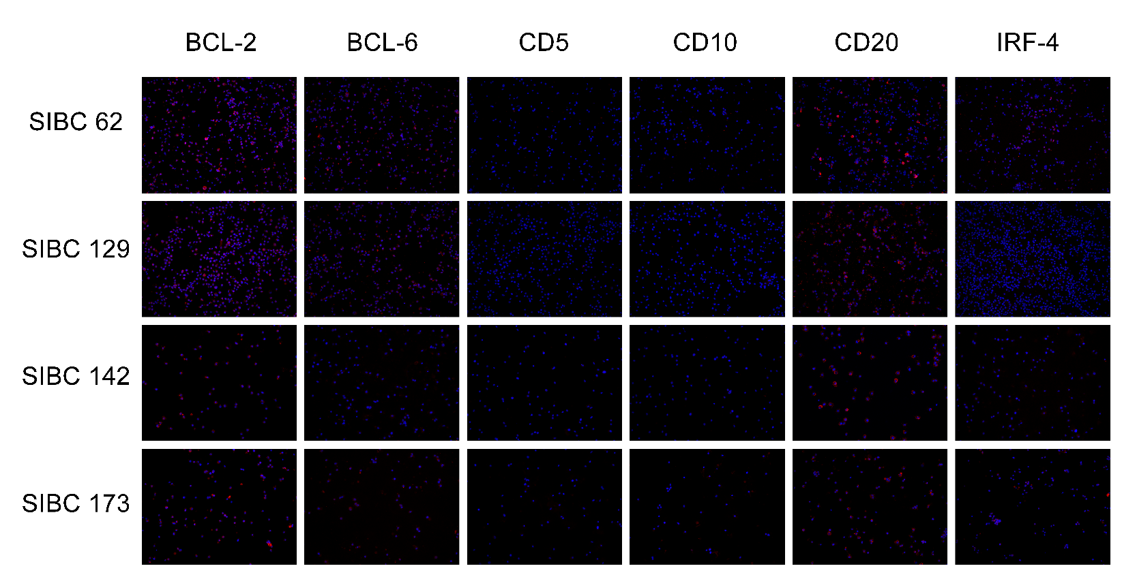

Supplement: S3 Fig — The immunophenotypes of SIBC-62, SIBC-129, SIBC-142 and SIBC-173 are shown by Immunofluorescence. (TIF) [file pone.0217161.s003.tif]

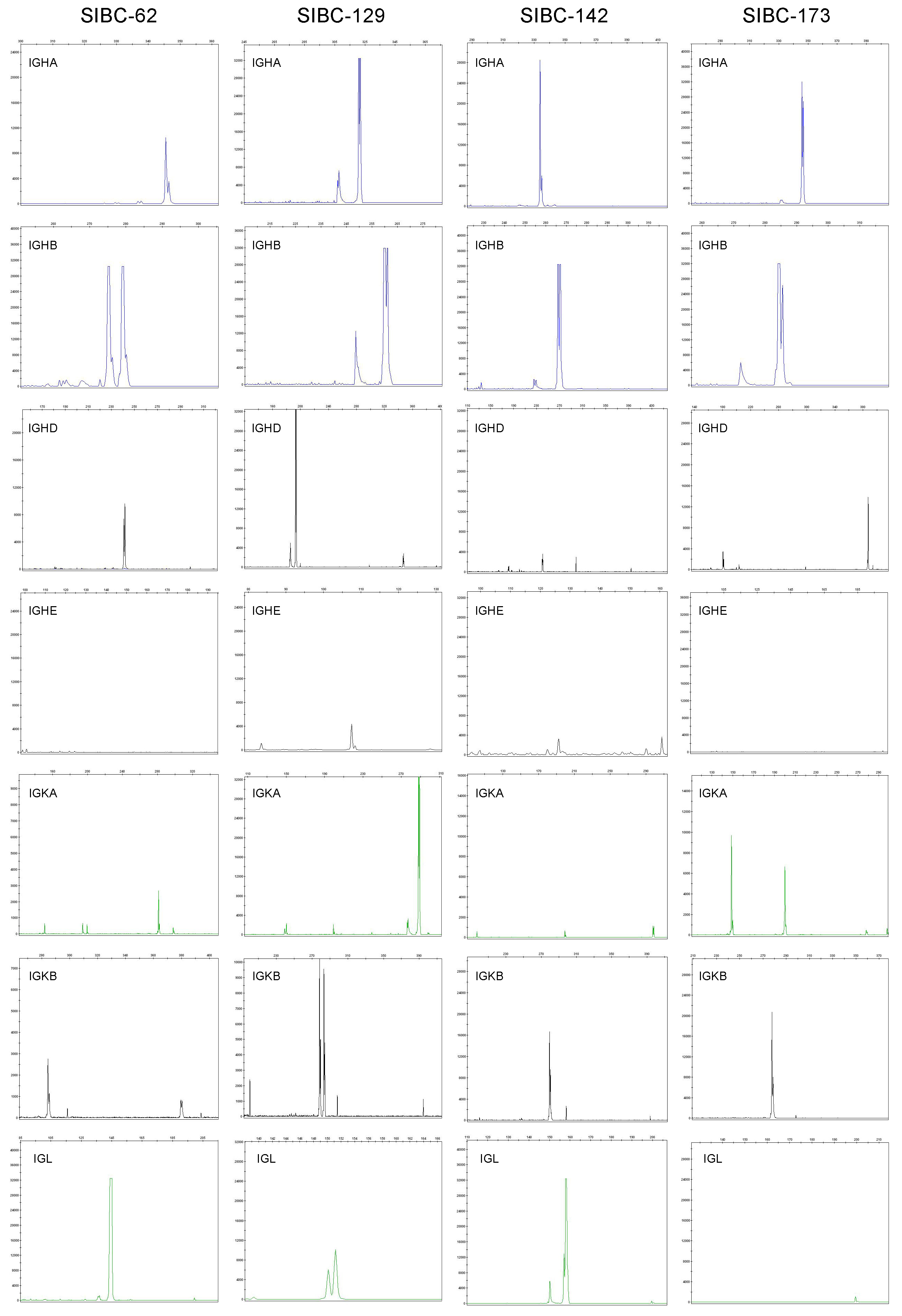

Supplement: S4 Fig — The Clonality of SIBC-62, SIBC-129, SIBC-142 and SIBC-173 are shown by Ig gene rearrangement. (TIF) [file pone.0217161.s004.tif]
